# Increased Biomass Yield of *Lactococcus lactis* by Reduced Overconsumption of Amino Acids and Increased Catalytic Activities of Enzymes

**DOI:** 10.1371/journal.pone.0048223

**Published:** 2012-10-25

**Authors:** Kaarel Adamberg, Andrus Seiman, Raivo Vilu

**Affiliations:** 1 Tallinn University of Technology, Department of Chemistry, Tallinn, Estonia; 2 Competence Centre of Food and Fermentation Technologies, Tallinn, Estonia; 3 Tallinn University of Technology, Department of Food Processing, Tallinn, Estonia; Lawrence Berkeley National Laboratory, United States of America

## Abstract

Steady state cultivation and multidimensional data analysis (metabolic fluxes, absolute proteome, and transcriptome) are used to identify parameters that control the increase in biomass yield of *Lactococcus lactis* from 0.10 to 0.12 C-mol C-mol^−1^ with an increase in specific growth rate by 5 times from 0.1 to 0.5 h^−1^. Reorganization of amino acid consumption was expressed by the inactivation of the arginine deiminase pathway at a specific growth rate of 0.35 h^−1^ followed by reduced over-consumption of pyruvate directed amino acids (asparagine, serine, threonine, alanine and cysteine) until almost all consumed amino acids were used only for protein synthesis at maximal specific growth rate. This balanced growth was characterized by a high glycolytic flux carrying up to 87% of the carbon flow and only amino acids that relate to nucleotide synthesis (glutamine, serine and asparagine) were consumed in higher amounts than required for cellular protein synthesis. Changes in the proteome were minor (mainly increase in the translation apparatus). Instead, the apparent catalytic activities of enzymes and ribosomes increased by 3.5 times (0.1 vs 0.5 h^−1^). The apparent catalytic activities of glycolytic enzymes and ribosomal proteins were seen to follow this regulation pattern while those of enzymes involved in nucleotide metabolism increased more than the specific growth rate (over 5.5 times). Nucleotide synthesis formed the most abundant biomonomer synthetic pathway in the cells with an expenditure of 6% from the total ATP required for biosynthesis. Due to the increase in apparent catalytic activity, ribosome translation was more efficient at higher growth rates as evidenced by a decrease of protein to mRNA ratios. All these effects resulted in a 30% decrease of calculated ATP spilling (0.1 vs 0.5 h^−1^). Our results show that bioprocesses can be made more efficient (using a balanced metabolism) by varying the growth conditions.

## Introduction

*Lactococcus lactis* is widely used in food- and biotechnological processes because the American Food and Drug Administration (FDA) classified it as GRAS in the Food Additives Amendment of 1958. Industrially, it is used in dairy starter cultures and there have been several attempts to use lactococci as cell factories for the production of various compounds (flavors, bacteriocins, vitamins etc.) [1]. Moreover, genetically modified strains of lactic acid bacteria are potential candidates for the production of orally delivered and live vaccines [2] as well as for the production of recombinant proteins because the secretion mechanisms and protein extraction procedures are less complicated when compared with Gram-negative bacteria [3].

The application of *L. lactis* in biotechnological processes is complicated by the requirement of complex growth media. Several amino acids such as glutamate/glutamine/arginine, histidine, isoleucine, leucine, methionine, and valine are essential for the growth of *L. lactis* IL1403 [4], [5]. Cocaign-Bousquet *et al.* report that serine and threonine are also necessary for minimal growth of this strain [6]. Based on an annotated network taken from the BioCyc database collection, only four amino acids cannot be synthesized by *L. lactis* IL1403, however, no published experimental results confirm this statement. Moreover, only a few of the 20 proteinogenic amino acids can be excluded from the growth medium without a reduction in growth yield and growth rate [6]. Despite much effort, the highest growth yields and growth rates obtained in defined media containing all amino acids remain 15 to 30% lower than those obtained in complex media such as M17 [7]. This can be explained by additional cofactors (vitamins) or nucleosides present in the M17 medium in addition to more efficient transport of peptides in M17 compared to amino acid transport in defined media [8]. Lactococci do not have specific amino acid transporters for each amino acid which can cause affinity dependent transport of specific amino acids. For example, Poolman and Konings [4] show that the uptake Km for valine is 12 µM while the Km for leucine is 6 µM despite the fact that both amino acids are transported by the same permease.

The use of defined media aids in the interpretation and reproducibility of metabolic studies, however, the fastidious and complex nutritional requirements of *L. lactis* complicate the design of defined media for fast and high growth yields. Usually, growth of lactococci is wasteful with respect to substrate use. In fermentative growth, up to 95% of carbon utilized by *L. lactis* can be used for energy generation by directing it into byproducts. Similarly, the nitrogen source is not solely used for biomass synthesis and typically a large proportion of amino acids remain unconsumed in the medium after growth [4], [7]. Progress in media optimization was accomplished by Zhang *et al* [5] who achieved biomass yields that were twice as high in medium containing a similar total amount of amino acids with different ratios than CDM media [4]. A new medium with lowered amino acid concentrations suitable for continuous cultivation of lactococci was developed in our laboratory to study the growth space of this organism at different specific growth rates [9]. The amino acid metabolism of lactococci and lactobacilli has been widely studied with respect to flavor development in cheese and other dairy products. Several amino acid pathways and regulation mechanisms have been analyzed and their potential roles in ATP synthesis, NAD^+^ regeneration etc. have been postulated [10]–[12]. However, a systematic analysis of the consumption strategies of amino acids in relation to the central metabolism (carbon, nitrogen and ATP spillage) using a quantitative multi-omics (systems biology) approach has not been published yet. Although genomic research and “omics” methods have made enormous progress during the past decade, a number of metabolic puzzles that could be studied using these developments remain unsolved. Systems biology is moving from measurements of relative numbers of proteins, mRNAs, metabolites, and fluxes towards quantitative analysis and should facilitate a more detailed understanding of the relationships between metabolic and energetic demands, and cell regulation. Several methods have been developed and applied to quantify proteins and mRNAs in *E. coli* and mammalian cells [13], [14]. In addition, absolute proteome and transcriptome measurements of *L. lactis* have been reported at three specific growth rates using chemostat cultivation [15].

The aim of this study is to determine amino acid consumption strategies and relationships between the amino acid metabolism and other cellular processes in *L. lactis* IL1403 while the specific growth rate (μ) is increased from 0.1 to 0.55 h^−1^ under glucose-limited conditions. Relative proteome and transcriptome data from continuous A-stat cultivation [9] is reanalyzed in this study to quantify the amount of mRNAs and proteins in the biomass. The transcription/translation level regulation of mRNAs and proteins, and post-translational regulation of metabolic fluxes are analyzed by combining 549 protein content measurements and mRNA pairs from five different specific growth rates with 179 metabolic fluxes.

## Results and Discussion

Growth of *Lactococcus lactis* is only possible in the presence of an external supply of several nutrients such as sugars, amino acids, vitamins and nucleobases, whose availability (content and proportions) determine the cell physiology and specific growth rate. To elucidate the quantitative relationships between mRNA/protein and protein/flux levels to the change of specific growth rate, a triple omics approach was used. Using the APEX method [13] protein abundances were calculated based on previously collected iTRAQ spectra for 549 proteins [9], covering 23% of the total open reading frames in *L. lactis* IL1403. In addition, Individual mRNAs were quantified based on DNA microarray intensities. Metabolic fluxes were calculated on the basis of a simplified metabolic network, which covers the central pathways, amino acid metabolism and biomonomer synthetic fluxes for nucleic acids, peptidoglycan, and lipids (SOM 1). Together with a quantitative proteome, metabolic fluxes allowed us to calculate the apparent catalytic activities of enzymes, costs for different metabolic routes and co-regulation coefficients used to describe regulatory patterns. With an increase in specific growth rate from 0.1 to 0.5 h^−1^, the growth efficiency (biomass yield) of *L. lactis* 1403 was increased from 0.10 to 0.12 C-mol biomass per C-mol of total carbon consumed. This increase was achieved by rearrangement of amino acid consumption patterns and a 3-fold increase in the apparent catalytic activities of enzymes and ribosomes. No pronounced reorganization of the proteome was observed, however.

### Protein distribution

**Figure 1:**
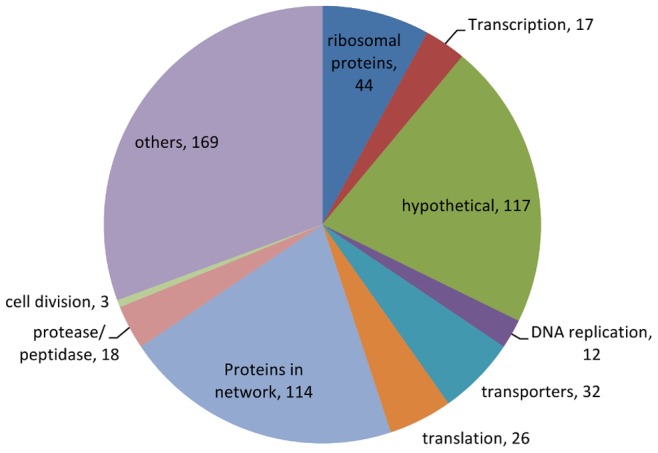
Distribution of proteins. All proteins quantified at all specific growth rates has been divided into different groups by function. Proteins in the network covers enzymes that are involved in the reactions used for metabolic flux analysis and listed in the File S1, Table S1. Lists of all proteins can be seen in the File S2, Table S8.

**Figure 2:**
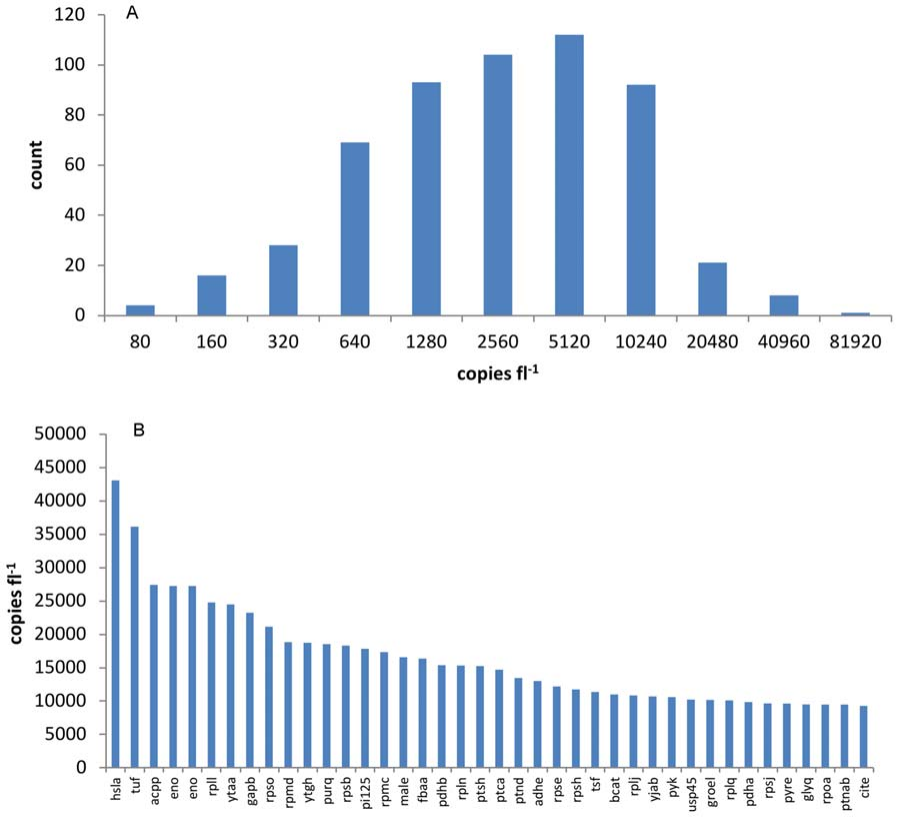
Distribution of protein concentrations. Concentrations (copies fl^−1^) of proteins at specific growth rate 0.2 h^−1^ are shown. Lower picture shows top 40 proteins fl-1 at the same specific growth rate.

Quantitative protein content measurements were carried out for 549 proteins, covering 23% of the total open reading frames in *L. lactis* IL1403. These include 114 proteins from the metabolic network (see Materials and Methods) and 70 proteins involved in translation (44 ribosomal, 22 aminoacyltransferase and 4 elongation factor proteins, **Figure 1**). A large number of these proteins belong to other enzymes or regulators not present in the metabolic network (168), 17 proteins for transcription, 12 for DNA replication, 18 for proteases/peptidases, and 32 proteins for other transporters. A significant number of proteins detected (117) could not be classified using information in the BioCyc database collection (www.biocyc.org).The abundance of individual proteins ranged from 50 to 48700 copies per fl (**Figure 2**) with the most abundant being translation factors (over 30000 copies fl^−1^), ribosomal proteins (giving in average 7300 to 9800 ribosomes fl^−1^ at a specific growth rate of 0.1 and 0.5 h^−1^, respectively), and glycolytic enzymes (over 7000 copies fl^−1^). High abundances were also observed for some stress factors (GroEL, GroES), membrane proteins (glucose Pts transporter and basic membrane protein Bmpa), and several proteins of unknown function. The copy numbers of enzymes involved in amino acid metabolism ranged from 300 to 15000 copies fl^−1^ while those of other proteins of biomonomer synthetic pathways remained below 4000 copies fl^−1^.

**Figure 3:**
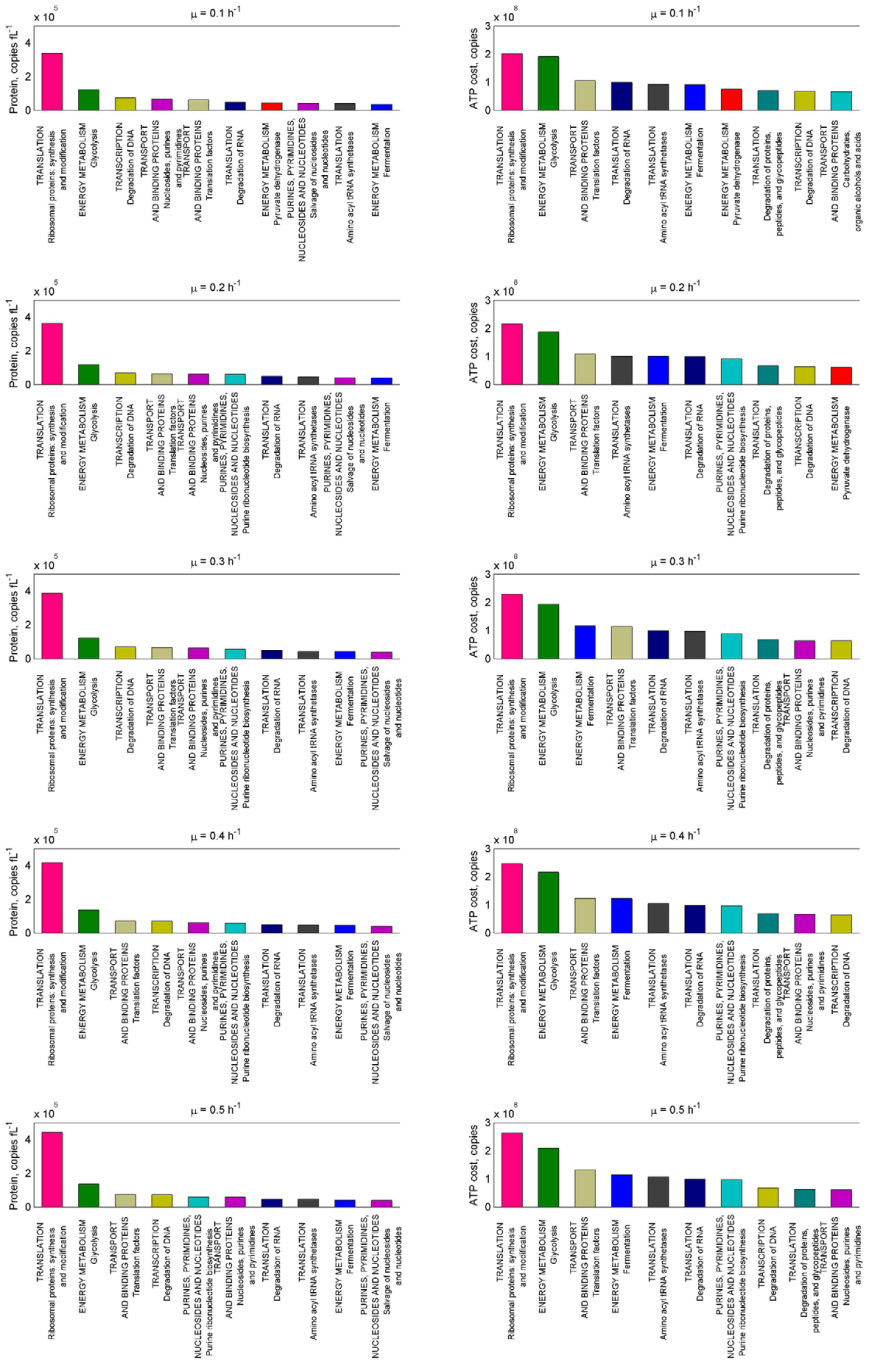
Changes of protein abundances and cost of ATP. Abundances are present in copies fl^−1^ and cost of ATP in ATP fl^−1^. Ten the most abundant pathways/cellular processes with increase of specific growth rate are illustrated. Distribution has been made according to the classification by Bolotin *et al* [23]. All data and distribution according to BioCyc database can be seen in the File S2, Tables S9 and S10.

The most abundant proteins function as part of the translation machinery (ribosomal proteins, aminoacyltransferases, elongation factors etc.) accounting for over 3.5*10^5^ copies fl^−1^ of individual protein chains in the cell from a total of 19.3*10^5^ protein molecules fl^−1^ (**Figure 3A**). However, ribosomal proteins are small and the amount of energy expenditure expressed as ATP spent for synthesis of ribosomal proteins and glycolytic enzymes were almost equal at a specific growth rate of 0.1 h^−1^ (2.0 and 1.9*10^8^ ATP fl^−1^, respectively, **Figure 3B**). All glycolytic enzymes account for up to 1.4*10^5^ copies fl^−1^ and the most abundant biomonomer synthetic pathway was nucleotide synthesis (up to 0.9*10^5^ copies fl^−1^). As the specific growth rate was increased, no significant re-organization of the proteome was observed, however. There were no up- or down-regulations larger than fivefold and only in 9% of the proteins up- or down-regulations were larger than two times.

### Carbon and nitrogen fluxes

**Figure 4:**
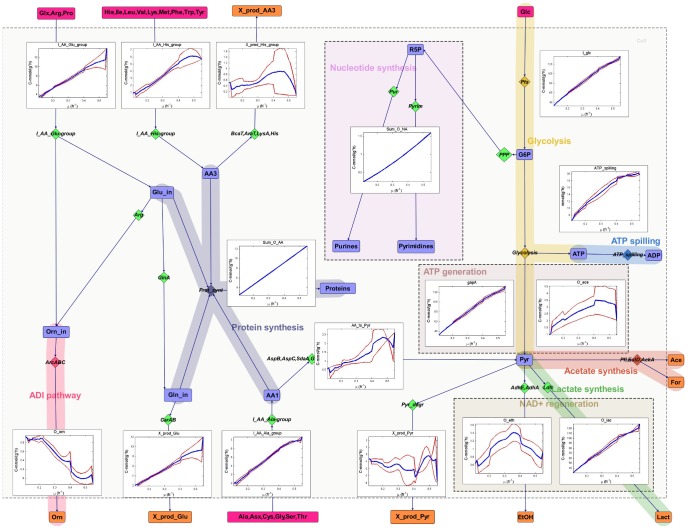
Simplified scheme of carbon flux rates. Fluxes are shown in C-mmol (gdw*h)^−1^ from A-stat experiments of *Lactococcus lactis*. Blue line represents average values of three independent experiments and red lines represent upper and lower values of standard deviations. Originally input values were experimentally measured at 20 time points and the other points were extrapolated between the measured values to calculate metabolic fluxes at interval of 0.01 h^−1^. Violet boxes are substrates, orange boxes are products and blue boxes intracellular metabolites. Diamonds illustrates proteins and pathways involved in the given conversion of metabolites. ace - acetate, lact - lactate, etOH - ethanol, Glc - glucose, Orn - ornithine, Glx - glutamate + glutamine, Asx - aspartate + asparagine, PPP - peptose phosphate pathway, Pyr - pyrimidine synthesis, Pur - purine synthesis, I_AA_Glu_Group - the sum of consumption of arginine, proline, glutamine and glutamate, I_AA_Ala_Group - the sum of consumption of alanine, asparagine, aspartate, cysteine, glycine, serine and threonine, I_AA_His_Group - the sum of consumption of histidine, isoleucine, leucine, lysine, methionine, phenylalanine, tryptophan, tyrosine and valine, X_prod_Pyr - unmeasured products to balance carbon in the calculations, X_prod_AA3 - unmeasured products from histidine, isoleucine, leucine, lysine, methionine, phenylalanine, tryptophan, tyrosine and valine to balance carbon in the calculations and X_prod_Glu - unmeasured products from arginine, proline, glutamine and glutamate to balance carbon in the calculations.

Simplified scheme of calculated carbon fluxes are given in **Figure 4**. Amino acid consumption pathways are summed up based on known degradation pathways from the BioCyc database. Consumption of amino acids that can be converted to pyruvate directly (Ala, Cys, Gly, Ser) or via oxaloacetate (Asx and Thr) are summed up, including degradation pathways through alanine transaminase (AspC), aspartate transaminase (AspB), cystathionine gamma-synthase (MetB2), serine hydroxymethyltransferase (GlyA), serine dehydratase (SdaA), and threonine degradation. Consumption of amino acids of glutamine group are merged together comprising arginine degradation to ornithine via the arginine deiminase pathway (ArcABC) and conversion of Glx or Pro to an unmeasured product from glutamine. Consumption of His, Ile, Leu, Lys, Met, Phe, Trp, Tyr and Val are also summed up because degradation pathways of these amino acids are not well characterized in *L. lactis* and no degradation products from these pathways have been measured. Details of the network are given in File S1, Table S1 and all calculated and measured fluxes are shown in File S1, Tables S2, S3, S4 and S5 and **Figure S1**.The list of protein names are provided in File S2.

The total carbon consumption rate increased from 31.7 to 177 C-mmol (gDW*h)^−1^ with an increase of the specific growth rate (μ) from 0.1 to 0.5 h^−1^. Most of the carbon was converted to lactate at a rate of 113 C-mmol/(gdw*h) while products from the mixed acid fermentation (acetate and ethanol) did not exceed 5 C-mmol (gdw*h)^−1^ (**Figure 4**). Calculated CO_2_ production comprised less than 1% of the total C-flux and the difference between the consumption of substrates and the formation of products (carbon shortage) from glycolysis was, on average, 1.6% (2.6 C-mmol gDW^−1^). This discrepancy may be explained by measurement errors of product formation (lactate etc.). Amino acid consumption comprised 16% of the total carbon influx at all growth rates from which 40% and 51% were used for protein synthesis at μ = 0.1 and 0.5 h^−1^, respectively. The remainder of the amino acids was used for nucleotide and aminosugar synthesis or converted to pyruvate or other excreted compounds not measured in this study. The consumption patterns of amino acids in *L. lactis* can be classified into three groups.

The first group is the degradation of overconsumed Ser, Ala, Asx, Gly, Thr and Cys into pyruvate. The total consumption rate of these amino acids increased from 1.4 to 7.0 C-mmol (gdw*h) ^−1^ (at μ = 0.1 and μ = 0.5 h^−1^, respectively) with the fastest rate of degradation to pyruvate occurring at μ = 0.41 h^−1^ (1.7 C-mmol gdw*h)^−1^ followed by a stable rate of overconsumption. Overconsumption of the pyruvate directed amino acids is possibly required to synthesize ATP, regenerate α-ketoglutarate, or balance glycolysis. The balancing of glycolysis by amino acids is required if there is not enough pyruvate to provide precursors for fatty acids and nucleotides, and to regenerate NAD^+^. The gap between pyruvate requirements for biosynthesis and regeneration of NAD^+^ was provided by overconsumption of Ser, Asx, Thr, Cys, Ala.

The second group are amino acids of the glutamate family - Arg, Glu, Gln and Pro whose consumption rates increased up to 11.5 C-mmol (gdw*h)^−1^ at μ = 0.55 h^−1^ from which about two thirds (9.2 C-mmol (gdw*h)^−1^ are wasted to form unknown products from glutamine. The reasons of this wasting are not clear as the knowledge of glutamate (2-oxoglutarate) degradation mechanisms to central metabolic products (glycolysis) in lactococci is limited. There are annotated genes from citrate to 2-oxoglutarate in the BioCyc database, however, expression of these genes and proteins were not observed. In addition, degradation products from Gln/Glu were not detected with the chromatographic methods used in this study. The third group consists of the remaining amino acids (Ile, Leu, Val, Phe, Trp, Tyr, Lys, His and Met). The degradation pathways of these amino acids are not well characterized in lactococci, however, the formation of hydoxy- and oxoacids from branched chain and aromatic amino acids is hypothesized to be involved in NAD^+^ regeneration or ATP production [12]. The sum of the third group of amino acids forms up to 6 C-mmol (gdw*h)^−1^ of which less than 15% were degraded to unmeasured products.

Specific fluxes did not change in proportion to the increase in specific growth rate at higher than μ = 0.4 h^−1^, excluding the specific rates of nucleotide and protein synthesis (**Figure 4**). The absence of an increase in specific fluxes was most pronounced for the overconsumption of amino acids (pyruvate from Ala group and unknown products from His group amino acids). This indicates that fewer amino acids were required for biosynthesis, additional ATP production, or NAD^+^ regeneration at higher growth rates. Although consumption rates were not proportional to specific growth rates, amino acid requirements for protein synthesis were met near the maximal specific growth rate by the consumption of external amino acids, thus suggesting a well-balanced amino acid metabolism. Consumption of glutamine, asparagine and serine exceeded the requirements for protein synthesis as glutamine and aspartate (synthesized from Asn) are amino group donors for nucleotide and amino sugar synthesis, and aspartate and glycine form the skeleton of purines. The medium used in this study did not contain aminosugars and all nucleotide precursors. Taking into account all reactions that transfer amino groups required for the synthesis of nucleotides and aminosugar, the minimal theoretical carbon wastage on the medium used would be 4.4 C-mmol gdw^−1^ Glu/Gln and 1.0 C-mmol gdw^−1^ Asp at μ = 0.55 h^−1^. Consequently, lactococci synthesize purines at a high cost with an added consequence of causing imbalances in both carbon and nitrogen metabolism. Stoichiometric imbalances result in low biomass yields, especially at low specific growth rates.

As nitrogen wasting decreases, energy (ATP) spilling also decreases. Energy spilling is calculated as the difference between total ATP produced and total ATP spent for synthesis of biomonomers with subsequent polymerization to macromolecules. Processes or reactions that may need additional ATP expenditures during cell growth, or are part of the maintenance costs such as the turnover of macromolecules, futile cycles, maintenance of membrane potential, pH homeostasis etc. [16], were not measured in this study. ATP spilling covered 46 to 35% of the total ATP production (μ = 0.1 and 0.55 h^−1^, respectively) and reached a maximum of 20 mmol (gdw*h)^−1^ at μ = 0.4 h^−1^, indicating more efficient growth at higher specific growth rates. More efficient growth was reflected in an increase in growth yield from 5.0 to 6.7 gdw mol-ATP^−1^. However, a higher ATP spilling rate in growing cells compared to that in non-growing cells (6 mmol (gdw*h)^−1^, estimated by the linear extrapolation of the specific ATP production curve) suggests that the maintenance-associated ATP costs are not constant in different growth phases. Also, the results show that more than 95% of ADP recycling to ATP is carried out through glycolysis and less than 5% of the conversion is taking place via secondary ATP production pathways (acetate production or carbamoylphosphate degradation). To estimate the efficacy of these energy generation pathways, a coefficient Eatp was calculated. Eatp was defined as the ratio of the total ATP produced from ADP in the given pathway to the ATP expenditure for the synthesis of enzymes of the pathway. We calculated that Eatp ratios were positive for both the acetate and ADI pathways (42 and 87 mol mol^−1^ i.e., mol-ATP produced per mol-ATP spent for the synthesis of enzymes of the pathways, respectively) indicating that these pathways could be induced to support ATP synthesis at low (below 0.2 h^−1^) specific growth rates. The largest Eatp value was observed for glycolysis (101 mol mol^−1^).

### Regulation of metabolism

Protein and mRNA abundance, as well as metabolic fluxes (where possible) were analyzed individually to find regulation patterns of transcriptional, translational, or post-translational control for each gene expression under glucose-limited steady state conditions. Median mRNA and protein values at μ = 0.5 h^−1^ were 2.4 and 1890 copies fl^−1^, respectively. The median ratio of protein to mRNA (pm) is thus 773, calculated from 549 pairs of mRNA and protein content measurements. This pm ratio was used to describe the amount of proteins produced per mRNA. The impact of transcriptional or translational regulation was shown by covariation coefficients between the specific growth rate and pm. At a covariation coefficient of zero, pm is kept constant at all μ values, which is characteristic for transcriptionally regulated genes because at all specific growth rates the same number of proteins is synthesized per mRNA transcribed. In total, 68 gene and protein pairs were observed with a covariance between μ and pm statistically close to zero (‘zero’ group), (gene statistics are provided in **Figure S2**). This group includes genes from the pentose phosphate pathway (Gnd, Zwf, Tkt) and DNA polymerase complex (PolC, GyrAB, TopA; DnaEJ), and single genes from the other pathways and processes.

**Figure 5:**
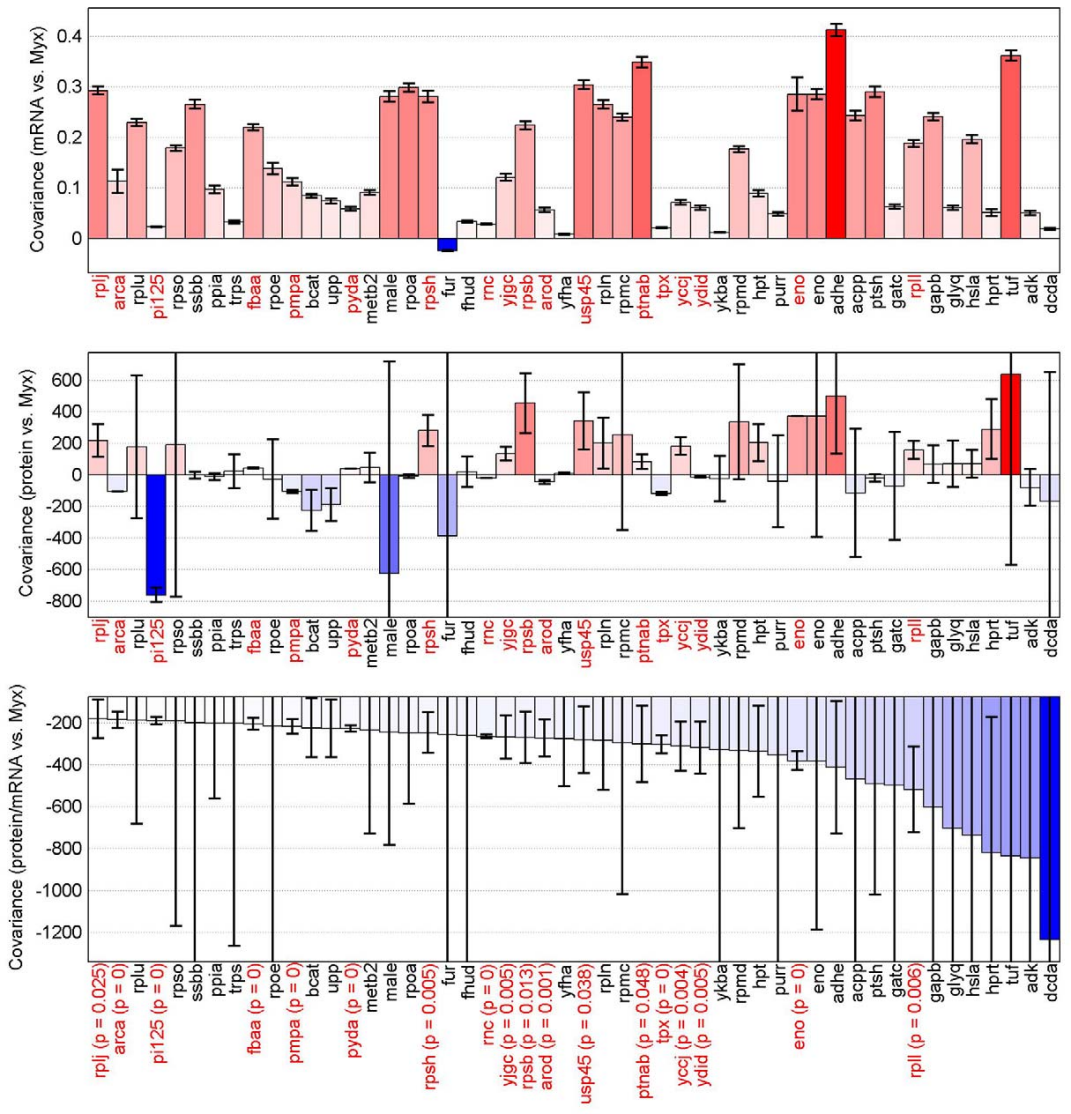
Regulation patterns between mRNA-s and proteins. Covariation coefficients between specific growth rate and mRNA, protein and pm (protein to mRNA ratio) ordered from top to down. Genes are sorted according to the covariance between specific growth rate and pm in decreasing order from left to right. Negative covariance between specific growth rate and pm is indicating that genes are regulated post-transcriptionally as the translation rate is decreasing compared to transcription rate i.e. there is less protein per mRNA with the increase of specific growth rate. Here are presented 50 most pronounced post-transcriptionally regulated genes. Red gene labels indicate that negative values of covariance between pm and specific growth rate are statistically significant (see the details of statistical hypothesis testing and P-value calculations in Materials and Methods). All covariation coefficients can be seen in **Figure S2**.

Posttranscriptional regulation is characterized by a negative covariation coefficient between μ and pm (**Figure 5**). Posttranscriptional regulation was observed for 142 genes (‘stat’ group), found to have statistically non-zero values for the covariation coefficients between pm and μ. Examples of posttranscriptional regulation include 13 ribosomal proteins and other proteins involved in translation (Tsf, ArgS, Gltx, LysS, MetS, TyrS), enzymes of pyrimidine synthesis (PydA, PyrCEHZ), ornithine-glutamate pathway (ArgEB), asparagine synthetase (AsnBH) and amino acid degradation (AraT, ArcA). Only four genes were characterized as translationally regulated (positive covariation coefficient between μ and pm). In these cases the translation rate lags behind the transcription rate increase on increase of specific growth rate. In other cases (285) the measured data did not pass the statistical tests (marked black in **Figure S2**).

It should be noticed that at μ = 0.5 h^−1^, post-transcriptional regulation was observed for the most abundant proteins in the cells: ribosomes, glycolytic enzymes, and proteins of glutamine and purine/pyrimidine synthesis pathways. In these cases, protein and mRNA abundances of individual gene products exceeded 3000 and 3 copies fl^−1^, respectively. Comparison of transcriptionally and post-transcriptionally regulated genes (68 ‘zero’ group and 142 ‘stat’ group genes, respectively) it is seen that the ‘zero’ group consists of longer proteins (52.9 vs 42.7 kD) and proteins with lower concentrations (1707 vs 3806 proteins fl^−1^) at μ = 0.5 h^−1^. The noted selectivity can be related to energy costs as more ATP is spent for the synthesis of longer proteins, and hence more ATP can be saved if transcriptional regulation was used [17]. This hypothesis is reasonable because space is limited within cells, so for enzymes with a high copy number, it may be reasonable to increase their catalytic activities instead of further increasing their copy numbers at increasing growth rates.

**Figure 6:**
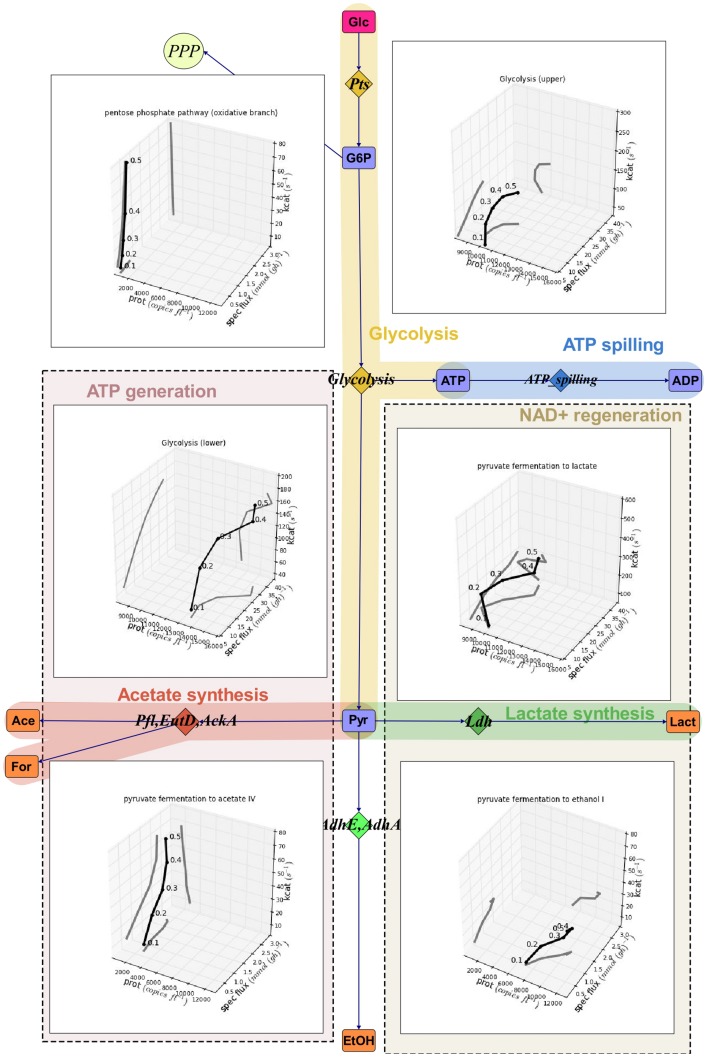
Multidimensional analysis of flux rates in A-stat cultures of *Lactococcus lactis*. Analysis is based on protein abundance (prot, copies fl^−1^) and apparent catalytic activities (k_cat_, s^−1^). In the three dimensional figures the average line and all three 2D projections are shown. The average values of proteins, specific fluxes and apparent catalytic activities are calculated based on all proteins present in the given pathway. Symbols are the same as described in the legend of Figure 4. More detailed picture can be seen in the **Figure S3** and interactive figures including individual proteins of different pathways are available through the supplementary link.

The metabolism of *L. lactis* over the range of specific growth rate 0.1 to 0.55 h^−1^ was studied in this work. To achieve higher specific flux rates cells should increase the abundance of the relevant enzymes or increase their catalytic activities. Proteome measurements showed that growth efficiency was more likely controlled by an increase in the catalytic activities of the enzymes (**Figure 6** and **Figure S3**) i.e., on average, the amounts of central metabolic enzymes or biosynthetic enzymes increased by 1.3 times, however, catalytic activities increased by 3.6 times, comparing μ = 0.1 and 0.55 h^−1^. Because protein complexes have not yet been annotated for lactococci, catalytic activities of enzymes were calculated per protein chain present in the complex, which can be termed apparent catalytic activity (k_cat_, s^−1^, ratio of specific flux to absolute amount of protein chain in biomass). The apparent k_cat_ shows an average throughput of molecules per protein chain. This value is typically smaller than the maximal enzymatic rate as measured by in-vitro enzyme assays. Based on the proteome of *L. lactis,* apparent k_cat_ values were very low at μ = 0.1 h^−1^ (below 10 s^−1^), including ribosomes (3.5 s^−1^), however, this excludes glycolytic enzymes (10 enzymes, k_cat_ 26 to 109 s^−1^) and lactate dehydrogenase (49 s^−1^), which are responsible for central carbon and energy metabolism. Proteins involved in translation (ribosomes, aminoacyltransferases) and biomonomer synthesis make up about half of the total protein abundance. In the already crowded cytoplasm this should limit the possibilities to increase the amounts of these proteins. Therefore, the cells maintained a high abundance of the proteins required for reproduction of the biomass, and the increase in growth efficiency can be largely attributed to an increase in the catalytic activities of proteins. Accordingly, the apparent catalytic values of enzymes over 20 s^−1^ were observed close to the maximal specific growth rate also for enzymes in the acetate/formate pathway (Pfl, EutD, AckA), pentose phosphate pathway (Gnd, Zwf), and for the glutamine transporter (GlnQ).

It was observed that the abundances of the first enzymes in the metabolic pathways were low, and thus they had high apparent catalytic activities. High k_cat_ values could indicate allosteric regulation and a hierarchical regulation of pathways as shown also by Daran-Lapujade *et al.* [18]. In glycolysis, proteins with low abundance (high apparent k_cat_) were phosphoglycerate kinase (Pgk), which is in the branch point of 3C compounds and glucose-6-phosphate isomerase (Pgi), which is in the branch point of C6 compounds in glycolysis. Lower concentrations in comparison to other glycolytic enzymes estimated by the enzymatic activities of these enzymes, have previously been observed [19]. Recently, Goel *et al.* [20] developed *in vitro* tests for measuring enzymatic activities that mimic *in vivo* conditions. They found similar ratios of enzymatic activities between glycolytic enzymes as obtained by Even *et al.* [19], however, with different absolute values. Therefore, *in vivo* like measurements of enzymatic activities should be combined with absolute proteome measurements to estimate the catalytic efficiency of enzymes *in vivo*. Not all pathways in *L. lactis* have been thoroughly investigated, however, a similar trend of regulation is likely because high k_cat_ values are observed, as noted, in the first enzymes of the pentose phosphate cycle (Zwf – G6P dehydrogenase, up to 54 s^−1^, average k_cat_ of this pathway was up to 25 s^−1^), purine synthesis (PurF, amidophosphoribosyltransferase, up to 1.7 s^−1^, average k_cat_ of pur operon was up to 1.2 s^−1^), and the second enzyme in pyrimidine synthesis (PyrC, dihydroorotase, 9.5 s^−1^, average k_cat_ of this pathway from carbamoyl-phosphate to oroditine-5-phosphate was up to 3.8 s^−1^, File S2, Table S9, columns AV to AZ). However, it cannot be excluded that k_cat_ values for functional protein complexes are different. In addition, very few studies have reported data about the protein complexes present in lactococci, a gap in the literature that should be addressed in the future.

**Figure 7:**
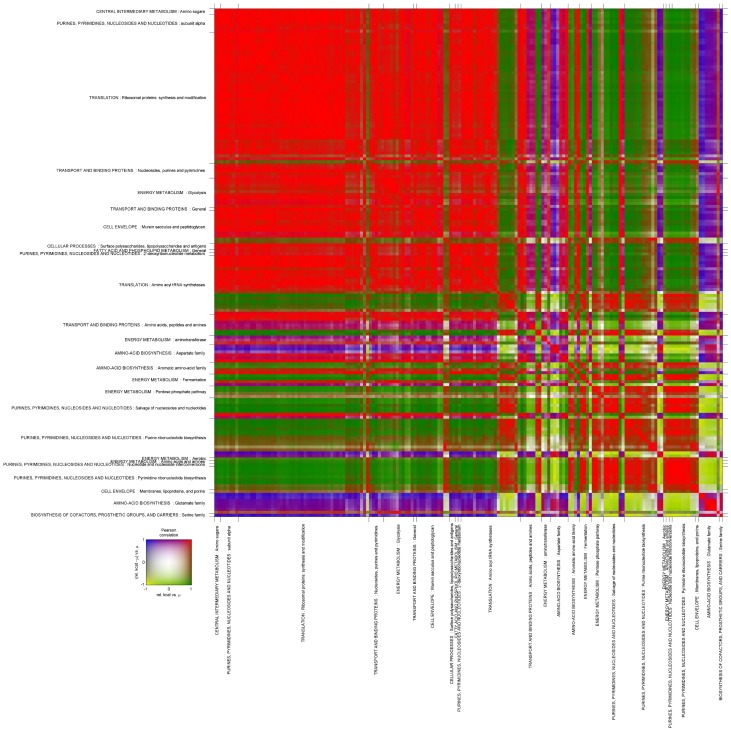
Two dimensional heat map of apparent k_cat_ values grouped. Grouping has been done according to the metabolic functions from Bolotin *et al* [23]. The small figure next to the heat map illustrates the colors of two dimensions corresponding to the correlation between apparent k_cat_ values of two genes (1st dimension, red or blue) or the correlation between the difference of relative apparent k_cat_ value and relative increase in specific growth rate of two genes (2nd dimension, green or yellow). Color in the crossing of two genes explains whether the k_cat_ values change in the same direction as the specific growth rate increases (red, green) or not (blue, yellow), and whether the changes in relative k_cat_ values are higher than the changes in relative growth rate (red, blue) or not (green, yellow).

Apparent k_cat_ analysis showed a clear relative change in the patterns of apparent k_cat_ values of different proteins in the pathways (**Figure 7**). Correlation analysis of these relative apparent k_cat_ dependencies can provide insight into the metabolic pathways or proteins that could operate together. If values of specific fluxes were increasing in proportion to the specific growth rate, we would expect them to increase five times as μ was increased from 0.1 to 0.5 h^−1^. Only 5 apparent k_cat_ values displayed this type of behavior. In most cases the increase in apparent k_cat_ values was between 3.5 and 4.5 times. Ribosomal proteins, aminocayltransferases, and glycolytic enzymes belonged to this group indicating that the rates of translation and energy supply should work with similar ratios at all growth rates. Regulation of ribosomal proteins should be very finely tuned because the apparent k_cat_ of ribosomal proteins increased by 3.75, on average, with a relative standard deviation less than 15% as μ was increased from 0.1 to 0.5 h^−1^. Proteins of fatty acid and murein synthesis also belong to this cluster, however, for different reasons. Because the surface to volume ratio of cells decreases by about 1.2 times with an increase in specific growth rate (μ = 0.1 to 0.5 h^−1^), the supply of cell wall components should decrease accordingly as the amount of enzymes in these pathways remained constant. The only enzymes with reduced apparent k_cat_ values belonged to the arg operon which explains the observed fast decrease in arginine overconsumption. Another cluster of enzymes active in purine and pyrimidine synthesis display an increase in apparent k_cat_ values over 6 times while μ is increased from 0.1 to 0.5 h^−1^. This may be caused by the relative difference in total RNA and proteome content in the biomass at different growth rates. Indeed, the amount of total RNA increased two times in the biomass while the proteome increased 1.7 times as μ increased from 0.1 to 0.5 h^−1^. A similar change in apparent k_cat_ values was observed for glutamine transport and synthesis (GlnA and GlnQ), however, these leveled off after μ = 0.35 h^−1^ concurrently with a balance in consumption of amino acids (see above).

The observed increase in apparent catalytic activities shows that post-translational regulation is an important mechanism for improving growth efficiency. High apparent k_cat_ values for glycolytic enzymes could be necessary to ensure a fast supply of ATP, however, a high abundance of enzymes responsible for synthesis of biomonomers at lower growth rates might be required for a quick response to changes in environmental conditions. If growth conditions improve, cells can rapidly increase the catalytic activities of enzymes without spending time and resources for the synthesis of new proteins. In the case of constant k_cat_, the amount of biosynthetic enzymes should be 5 times smaller at a specific growth rate of 0.1 h^−1^, compared with that at 0.5 h^−1^. The ATP costs for the synthesis of biosynthetic enzymes and corresponding transporters at 0.1 and 0.5 h^−1^ are 5.2*10^8^ and 5.8 *10^8^ molecules of ATP per fl, respectively. File S2, Table S9 provides the sum of ATP costs (column BJ) for production of proteins in the pathways of amino acid biosynthesis, biosynthesis of cofactors, cell envelope, fatty acid metabolism, purines and pyrimidines and transporters of amino acids and nucleosides (column A). These ATP costs were 22.1 and 24.2% from the total synthesis cost of proteins (23.7 and 24.0*10^8^ molecules of ATP per fl). However, if the proteins were having constant high catalytic activities, 4.8% would be sufficient for biosynthetic purposes at 0.1 h^−1^, meaning that 17.3% of the total cost for ATP biosynthesis could be saved. However, if the growth rate increased from 0.1 to 0.5 h^−1^, under these minimal conditions, the production of the roughly 20% extra proteins required for biosynthesis would take 16 minutes (one fifth of cell cycle time at 0.5 h^−1^) to produce, which could affect the adaptation of the cell to the new physiological state. To maintain the same amount of a given enzyme in the biomass, the protein synthesis rate must be proportional to the specific growth rate. When the specific growth rate is increased from 0.1 to 0.5 h^−1^ with a constant low k_cat_, it is clear that translation of the protein should be 25 times faster at μ = 0.5 h^−1^ than that at 0.1 h^−1^. In practice, increase in metabolic fluxes are almost equally balanced by an increase in translation rate (ribosomal k_cat_), and enzymatic activities increase by 3.7 times with a small observed increase in the amount of protein (less than 1.8 times).

### Conclusions

This study combines quantitative multilevel analyses (steady state cultivation with absolute flux, and apparent k_cat_ values of enzymes and abundances of mRNA and proteins) to analyze metabolic responses of *L. lactis* if the specific growth rate is increased from 0.1 to 0.5 h^−1^. It was shown that the biomass yield and the efficiency of ATP usage can be increased at high growth rates close to μ_max_ (0.5 h^−1^) (1) through rearrangements in amino acid metabolism, (2) by increasing translation efficiency, (3) by improving the catalytic activities of enzymes, and (4) by reducing the synthesis of non-critical proteins. Based on this knowledge, more detailed cell models can be created in which the proteomic and catalytic relationship between different cell processes are taken into account. In practice, this knowledge could enable one to optimize cultivation processes to achieve higher biomass yields of lactic acid bacteria by both the changing environmental conditions during cultivation and balancing the culture media to minimize the wastage of amino acids and other compounds.

## Materials and Methods

### Bacterial strain and culture media

*Lactococcus lactis* subsp. *lactis* IL1403 was provided by INRA (France). A defined cultivation medium, consisted of 20 amino acids, minerals and vitamins, and inoculum was prepared at 34°C for 20 h as previously described [9].

### Cultivation system

The cultivation system was the same as in [9] and run in A-stat mode. Shortly, the cultivation system consisted of a Biobundle 1.25 L fermenter (Applikon, The Netherlands) controlled by an ADI 1030 biocontroller (Applikon), and cultivation control program “BioXpert” (Applikon). All cultivation experiments were performed at 34°C, pH 6.4 and in an anaerobic (N_2_) environment using the A-stat algorithm: D = D_0_ + a_D_*t, where D_0_ is the initial dilution rate (h^−1^), a_D_ is the acceleration rate (h^−2^), and t is the time from the start of acceleration (h).

### Experimental data

Experimental data quantifying glucose and amino acid consumption and fermentation product formation rates were taken from Lahtvee *et al.* [9]. Transcriptome and preotome data, also from [9], were reprocessed to get absolute numbers for the content of mRNA-s and proteins. For mRNA abundances, average intensities from seven different mRNA oligos from a single experimental point in the Agilent transcriptome array (GEO number GSE26536) were summed, excluding ribosomal RNA (rRNA) and transport RNA (tRNA) units. This sum corresponds to the total amount of mRNA. It was assumed that mRNA composed 5% of total RNA (that was measured) and the intensity units of each mRNA were proportional to the total mRNA. The amount of molecules of each mRNA in the biomass was calculated taking into account the molecular weights of each mRNA. Proteome data were recalculated using the APEX algorithm (details of calculations can be seen in File S2, Table S11), which assumes proportionality of spectral counts of peptides from each protein to be linearly correlated to abundance of this protein in the cell [13].

### Metabolic network

A simplified metabolic network of *L. lactis* subsp. *lactis* IL1403 under different growth conditions was constructed based on information from BioCyc database (www.biocyc.org) and using a spreadsheet to perform the metabolic flux calculations. Only the reactions of the central pathways (glycolysis, pentose phosphate cycle, pyruvate metabolism), amino acid metabolism and biomonomer synthetic fluxes with branch-point metabolites were taken into account to construct the network (reaction list in File S1) i.e. all linear reaction chains were combined to a single enzymatic complex responsible for flux (altogether 103 merged pathways or single reactions). The metabolic system contains 40 independent fluxes (specified) and 63 dependent (calculated) fluxes. Of these 22 are substrate consumption fluxes, 5 are product formation fluxes and 36 are biomass production fluxes. Six of the independent fluxes were balance fluxes to calculate production of non-measured products such as ammonia (O_NH3) and CO2 (O_CO2), additional compounds (not identified in this study) from pyruvate (X_prod) and glutamate (X_glu), or to calculate ATP and NAD(P)H spilling (fluxes fbp and NADH_bal, respectively). Dependent fluxes (63) were consumption of glucose, amino acids (all proteinogenic amino acids plus ornithine) and hypoxanthine, production of organic acids (LAC, ACE, FOR) and ethanol, and fluxes for biomass synthesis. Steady state growth was assumed in the flux calculation and analysis. Reverse yields and rates of substrate/product consumptions/formations (μmol gdw-1 and μmol gdw-1 h-1, respectively) were calculated from processed HPLC/UPLC and OD measurements using BioXpert and in-house software (Python) for data splining (originally ∼20 measured points in the range of μ = 0.1 – 0.6 h^−1^ were measured). Arrays of the splined extracellular fluxes (each expressed per 0.01 h^−1^of dilution rate) were used as inputs for the calculation of the dependent fluxes in the spreadsheet provided (File S1). The average and standard deviations of each flux from three independent experiments and correlation/covariation coefficients (R) between the dilution rate and each flux and between each flux pair were also calculated using the spreadsheet.

The metabolic network contains 197 enzymatic proteins. Among those, the abundance of 114 enzymes (58%) were quantified using the APEX method, 24 proteins (12%) were quantified only at a relative level (mainly single enzymes from certain pathways, ex ArgJ, AroE, ProB, PurE, PyrF, TrpB etc) and 45 proteins (23%) could not be detected in the biomass (involved in histidine (10 proteins), branched chain amino acid (7) and aromatic amino acid (9) synthesis and single genes from certain pathways, ex. AccB, FabH, MurA1, PurN, Tmk etc). Histidine and branched chain amino acids are essential for the growth of *L. lactis* IL1403 (as shown by Cocaign-Bousquet [6] and Zhang [5]) and consumption of these amino acids meets the demand of biosynthetic requirements. It is possible that the absence of these proteins in the cells is a metabolic consequence because genes from his and leu operons were not expressed. Additionally, 14 hypothetical genes were added into the network that are not identified in KEGG or BioCyc databases for *L. lactis*, however, these functions are required for functional biosynthesis (transporters of organic acids, branched chain amino acids and hypoxanthine, undecaprenol kinase (2.7.1.66) in peptidoglycan, phosphatidylglycerophosphatase (3.1.3.27) in lipid and phosphoglycerol transferase (2.7.8.20) and N-acetyl-glucoseaminyl transferase (2.4.1.-) in lipoteichoacid synthesis and genes of exopolysaccaride production).

The biomass composition was obtained by reanalyzing published data [9]. The main difference is our more detailed analyses of the cell wall including lipoteichoacids in addition to the peptidoglycan and lipid layer (see calculations below). For MFA inputs, all consumption and production values were calculated per organic matter measured (plus minerals 7%) in the cell, giving an average biomass conversion factor of 0.32 g l^−1^ per 1 OD unit. Biomass composition was calculated as a function of specific growth rate for all biomass components on the basis of experimentally measured values. Total RNA was measured using a Qiagen kit, amino acids were measured after biomass hydrolysis using a UPLC ACCQTAQ kit, fatty acids were measured after biomass hydrolysis by UPLC [21], DNA content was measured using a Qiagen kit and total glucose equivalents were measured using the anthrone method and the ratio of the sum of acyl moieties to glycerol phosphate, and the ratio of the sum of acyl moieties to peptidoglycan units were taken from the literature [22] to calculate the amount of glycerol phosphate and peptidoglycan in the biomass. Based on experimental data, several simplifications were included to calculate the macromolecular composition of the cell. As fatty acids and glucose equivalents were determined experimentally, glycerol-P and the distribution of these monomers between LTA, PG, PS and lipids were calculated. Proportional fractions of summed acyl equivalents, glucose equivalents and glycerol-P in LTA, PS and lipids were taken from the literature [22]. Glycerol-P was calculated on the basis of the ratio of glycerol-P and total acyl moieties contained in lactococci from Oliveira *et al.* [22] (2.7 mol per mol). Similarily, PG sugars were calculated based on the ratio of NAG + NAM and sum of acyl moieties in lactococci [22] that was 1.77 mol per mol. The biomass monomer compositions at selected specific growth rates are presented in File S1, Table S6 and matrices of calculable and measured fluxes in the File S1, Table S7.

### Statistical analysis

Based on the abundance of mRNAs and proteins, together with metabolic fluxes, (1) the protein to mRNA ratio (pm, mol mol^−1^), and (2) the apparent catalytic activity of enzymes (k_cat_, s^−1^) were calculated as follows: 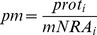
(1)where prot_i_ and mRNA_i_ are abundances of individual protein and mRNA in biomass (copies fl^−1^), respectively, and: 
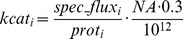
(2)where spec_flux_i_ is flux carried out by protein i (mol (g*s)^−1^), N_A_ is Avogadro number, 0.3 characterizes the part of dry mass in gram of cells and 10^12^ is the conversion factor from fl to g if density is 1 g ml^−1^.

Covariation coefficients between the specific growth rate and measured parameters (mRNA, protein abundance) and the calculated (flux, pm, k_cat_) parameters were calculated. Along with the calculation of covariance coefficients, their uncertainties were estimated. The uncertainty values of covariance coefficients were required for their statistical hypothesis testing in the regulation analysis.

### Uncertainty of covariance

Uncertainty of covariance was calculated as follows: 
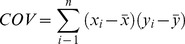
(3)


The relative uncertainty of covariance can be found from: 
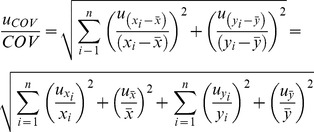
(4)


As mean values of 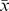 and 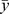 are calculated according to formulas 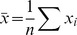 and 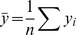, then the uncertainty of mean value can be found:



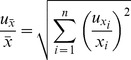
 and 
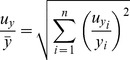
(5)


Therefore, the relative uncertainty of covariance is equal to: 
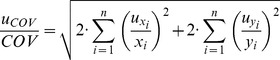
(6)


The uncertainty of the growth rate is considered to be zero as it was not estimated. Therefore, the member with *y* in the second half of the equation is zero.

As pm and k_cat_ are ratios between mRNA and protein or specific flux and protein, then the uncertainty of pm or k_cat_ is comprised of uncertainty estimations of members of a particular ratio. The uncertainty of specific flux was available only for a subset of the genes. Therefore, in these cases, we did not account for the uncertainty value of k_cat_, because otherwise the anslysis would give an unequal statistical preference to the genes for which the uncertainty of specific flux values was not estimated.

Therefore uncertainty estimations of pm and k_cat_ are follows: 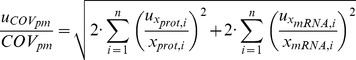
(7) and 
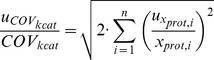
(8)


### Test of significance

Calculated covariance values were subjected to statistical hypothesis testing. First, the hypothesis that the absolute values of covariance are higher than zero at a statistically significant level was tested. A one sided z-test was applied to compare the absolute values of covariance to zero. Uncertainty estimation, calculated as described above, was used as a nuisance parameter. Genes with a significance level below 0.05 were considered statistically significantly different from zero.

The rest of the covariance values do not differ significantly from the zero. This group contains genes whose covariance value is zero. Additionally, genes with very high uncertainty values belong also to this group. To distinguish between covariance values that are actually equal to zero, an additional hypothesis test was applied. First, it was expected that in most of the cases, when the covariance value does not differ from zero at a statistically significant level, it is because the covariance value is actually zero and not because the uncertainty value of covariance is very high. Therefore, a one sided normal distribution was expected to be applicable for absolute values of covariance. A limit, corresponding to a significance level of 95%, was calculated according to this distribution. Next a z-test was again applied to test the hypothesis that absolute values of covariance are lower than this limit. A covariance value was considered to be zero if the value was below the limit at significance level below 0.05.

Eventually, genes were divided to three groups. One group corresponds to genes with covariance value statistically higher than zero. The second group corresponds to genes with covariance value equal to zero at a statistically significant level. The remainder is genes described by a level of covariance that is too high to determine what kind of regulation was occurring. Identical hypothesis testing was applied to covariance values of pm and k_cat_.

## Supporting Information

File S1 **List of reactions.** Reactions used for metabolic flux analysis and calculated values for individual experiments (Exp1, Exp 2, Exp3), along with average values of three independent experiments.
(XLSX)

File S2 **Omics data sets.** Data describing mRNA and protein abundances (molecules/fl), specific flux values (mmol (g*h) ^−1^), apparent catalytic activities of proteins (s^−1^), protein to mRNA ratio (mol/mol) and correlation and covariation coefficients between the specific growth rate and measured or calculated parameters.
(XLSX)

Figure S1 **Average metabolic flux values (blue line) with standard deviations (red lines) of three independent A-stat experiments with** ***Lactococcus lactis*** **IL1403.**
(JPG)

Figure S2 **Regulation patterns between mRNA-s and proteins.** Covariation coefficients between the specific growth rate and mRNA, protein and pm (protein to mRNA ratio) for each gene product organized from largest to smallest value. Genes are sorted according to the covariance between specific growth rate and pm. Blue gene labels indicate that the covariance between pm and specific growth rate for transcriptional regulation is not statistically significantly different from zero i.e., there are no changes in protein production per mRNA with an increase in specific growth rate. Red gene labels indicate that the covariance between pm and specific growth rate is statistically significant for post-transcriptional or translational regulation. Negative covariation between pm and specific growth is evidence of a post-transcriptional regulation because the translation rate is decreasing compared to the transcription rate. In contrast, a positive covariation demonstrates translational regulation because translation is more enhanced compared to transcription as the specific growth rate increases. Details of statistical hypothesis testing can be found in Materials and Methods.
(JPG)

Figure S3 **Multidimensional analysis of flux rates in A-stat cultures of** ***Lactococcus lactis*** **based on protein abundance (prot, copies fl** ^−1^ **) and apparent catalytic activities (k_cat_, s** ^−1^ **).** Symbols and pathways are the same as described in the legend of **Figure 4**. For the transport reactions and formation of unknown products, only 2D plots (specific flux vs specific growth rate) are shown as in the **Figure 4** because no proteome data is available for these pathways. **Supplementary link for multidimensional analysis of flux rates in A-stat cultures of** ***Lactococcus lactis***: https://sites.google.com/a/tftak.eu/3d-plots-lactococcus-lactis-plos-one-2012/adamberg-et-al-som-figures-increase-of-biomass-yield-of-lactococcus-lactis includes interactive figures of three dimensional plots between changes of absolute amounts of proteins (copies fl^−1^), specific fluxes (mmol (g*h) ^−1^) and apparent catalytic activities (s^−1^). Plots are drawn based on the data given in the File S2.
(JPG)
