# Supplementary figures and images for: Increased Biomass Yield of Lactococcus lactis by Reduced Overconsumption of Amino Acids and Increased Catalytic Activities of Enzymes
Source: PLoS One. 2012 Oct 25;7(10):e48223. doi: 10.1371/journal.pone.0048223 (PMC3485057; doi:10.1371/journal.pone.0048223)

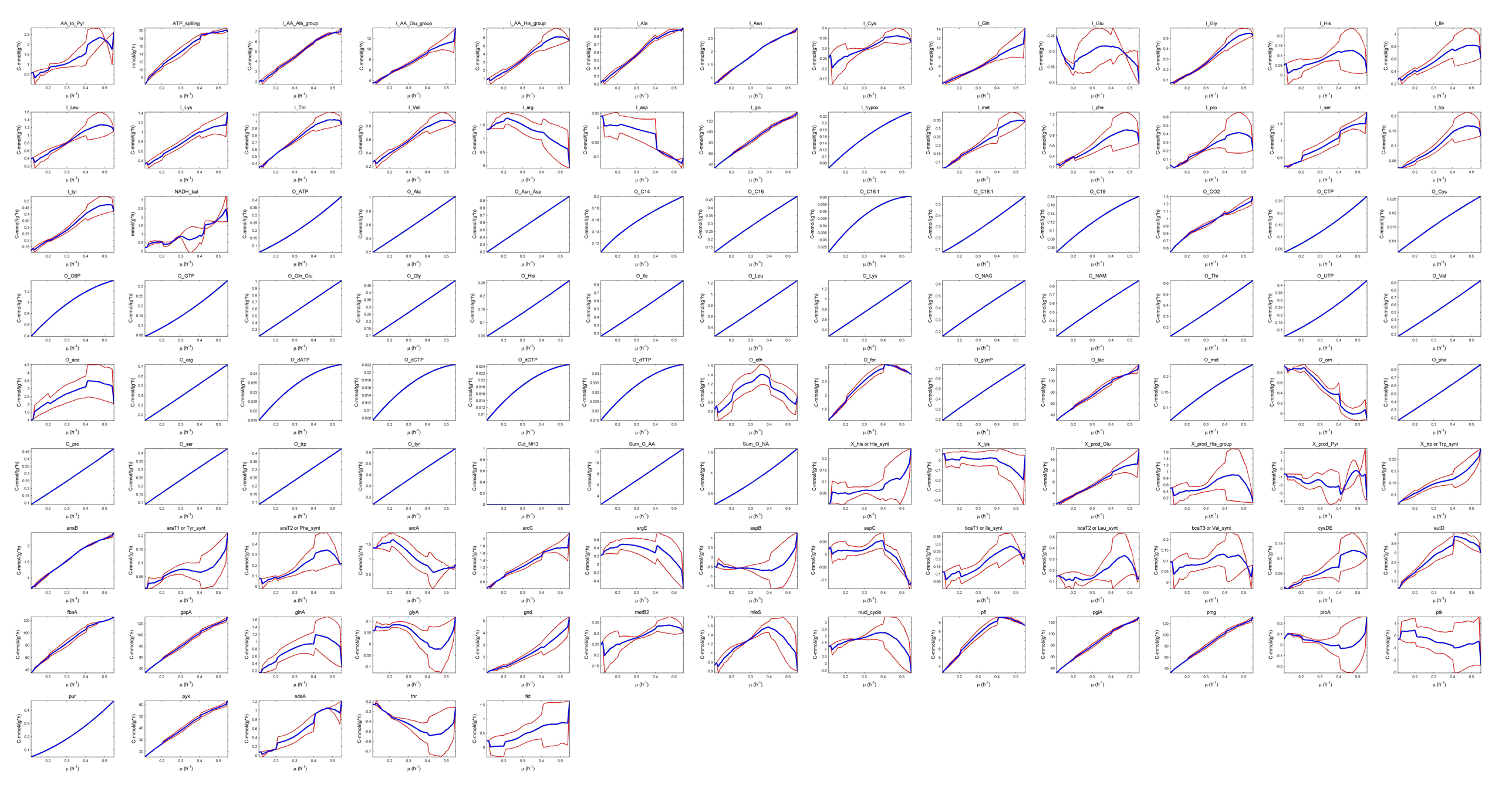

Supplement: Figure S1 — Average metabolic flux values (blue line) with standard deviations (red lines) of three independent A-stat experiments with Lactococcus lactis IL1403. (JPG) [file pone.0048223.s003.jpg]

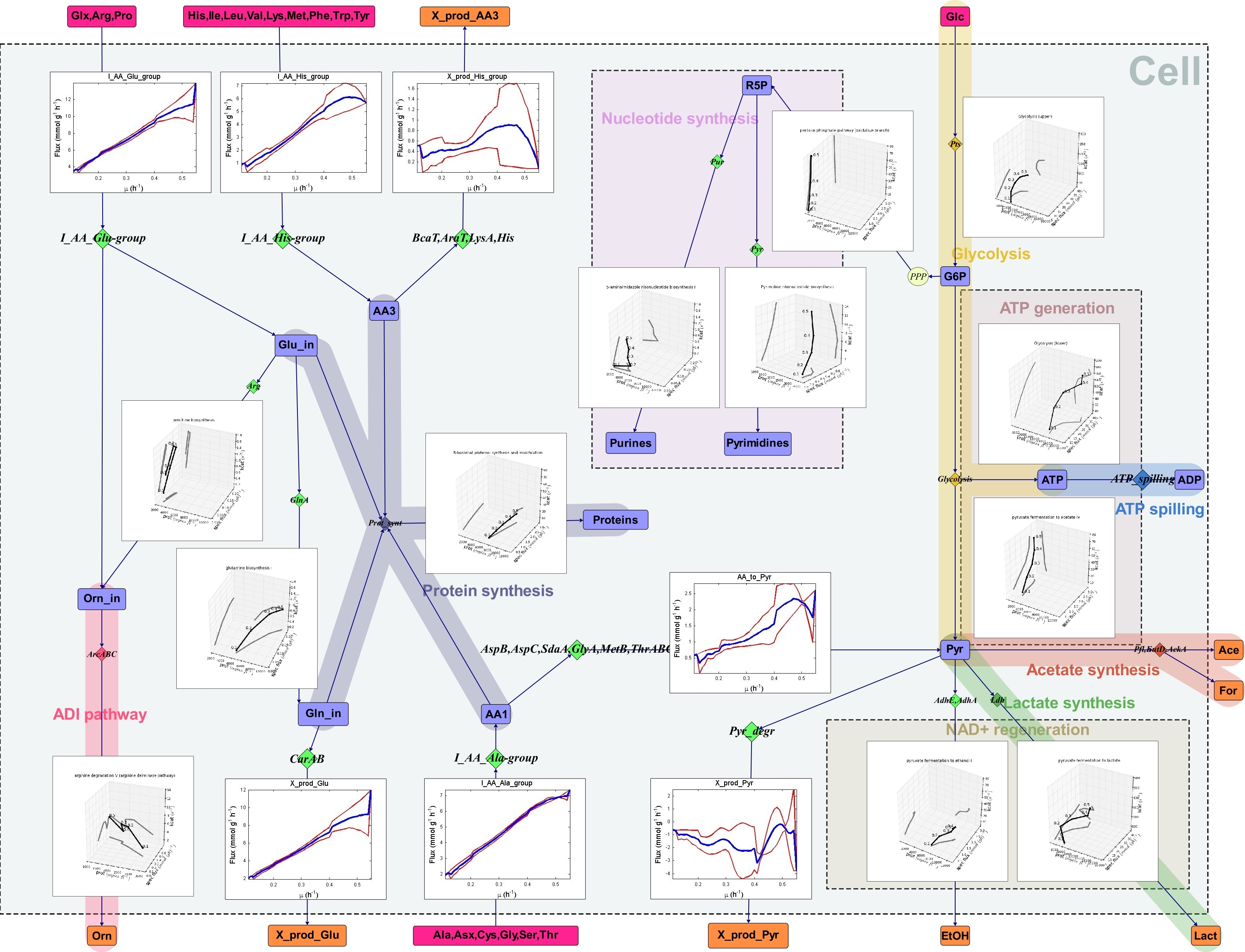

Supplement: Figure S3 — Multidimensional analysis of flux rates in A-stat cultures of Lactococcus lactis based on protein abundance (prot, copies fl −1 ) and apparent catalytic activities (kcat, s −1 ). Symbols and pathways are the same as described in the legend of Figure 4 . For the transport reactions and formation of unknown products, only 2D plots (specific flux vs specific growth rate) are shown as in the Figure 4 because no proteome data is available for these pathways. Supplementary link for multidimensional analysis of flux rates in A-stat cultures of Lactococcus lactis: https://sites.google.com/a/tftak.eu/3d-plots-lactococcus-lactis-plos-one-2012/adamberg-et-al-som-figures-increase-of-biomass-yield-of-lactococcus-lactis includes interactive figures of three dimensional plots between changes of absolute amounts of proteins (copies fl−1), specific fluxes (mmol (g*h) −1) and apparent catalytic activities (s−1). Plots are drawn based on the data given in the File S2. (JPG) [file pone.0048223.s005.jpg]
